# Environmental and microbial factors shaping SARS-CoV-2 RNA decay in wastewater: insights from batch tests and a lab-scale sewer pipeline simulator

**DOI:** 10.1038/s41598-026-44857-y

**Published:** 2026-03-19

**Authors:** JooAhn Jung, Lan Hee Kim, Sungpyo Kim, Hyun Sik Jun

**Affiliations:** 1 Department of Biotechnology and Bioinformatics, College of Science and Technology, Korea University, 2511 Sejong-ro, Sejong city, 30019 Republic of Korea; 2 Research Institute for Advanced Industrial Technology, Korea University, 2511 Sejong-ro, Sejong city, 30019 Republic of Korea; 3 Department of Environmental Systems Engineering, Korea University, 2511 Sejong-ro, Sejong city, 30019 Republic of Korea

**Keywords:** Wastewater-based surveillance, Viral RNA decay kinetics, Coronavirus RNA persistence, Lab-scale sewer system simulator, Human coronavirus NL63 surrogate, Environmental sciences, Microbiology, Water resources

## Abstract

Wastewater-based surveillance (WBS) can provide early warning of outbreaks, but wastewater RNA signals may be underestimated due to analytical limitations and in-sewer attenuation driven by matrix conditions and conveyance. Using human coronavirus NL63 (HCoV-NL63) as a BSL-2 surrogate to characterize coronavirus RNA decay (distinct from SARS-CoV-2), we quantified loss kinetics as a function of pH (2, 5, 7, 8), temperature (20, 30 °C), microbial abundance, suspended solids (SS; 74–216 mg L^− 1^), and transport distance. Batch tests showed that higher microbial concentrations markedly increased decay rates: in raw wastewater at 30 °C, the first-order decay constant *k* reached 2.21 d^− 1^, whereas filtration and/or microbial suppression reduced *k* to 1.12–0.47 d^− 1^. A lab-scale sewer pipeline simulator further showed faster decay with increasing transport distance, and faster decay in wastewater than in dechlorinated tap water at 25 °C (*k* = 0.52 vs. 0.28 d^− 1^). Across the conditions evaluated, microbially mediated processes were the dominant drivers of viral RNA loss. These decay kinetics provide a basis to interpret—and, where appropriate, adjust—SARS-CoV-2 wastewater RNA measurements across diverse environmental and conveyance conditions.

**Supplementary Information:**

The online version contains supplementary material available at 10.1038/s41598-026-44857-y.

## Introduction

Wastewater-based surveillance (WBS) is a cost-effective approach for monitoring community health by quantifying pathogens and other biomarkers in wastewater^1–4^. Before the COVID-19 pandemic, WBS has been used both to support poliovirus eradication through pathogen surveillance and to estimate illicit drug consumption in prisons and jails^2,3^. During the COVID-19 pandemic, WBS provided an aggregate, population-level indicator of SARS-CoV-2 infection burden by capturing viral shedding from infected individuals across multiple infection stages, including asymptomatic infections, within a given sewershed/catchment. Building on these established applications, WBS has expanded to include surveillance of additional infectious pathogens and chemical biomarkers (e.g., metabolites), broadening its utility for public health monitoring^5–7^.

SARS-CoV-2 RNA can persist longer than infectious virus in aquatic matrices, and field studies have reported continued RNA detection in wastewater for > 19 days after the end of a local outbreak (i.e., after no new confirmed cases were reported)^8,9^. With these substantial advantages, WBS was applied in many countries such as USA^10^, Hong Kong^11^, Denmark^12^, Netherlands^13^, Australia^14^, Qatar^15^, Finland^16^, South Africa^17^, Portugal^18^, South Korea^19^ to elucidate the regional or national COVID-19 outbreaks occurring in both symptomatic and asymptomatic populations.

To enable more systematic and complementary WBS, accurate and sensitive detection of pathogen-associated genetic material in wastewater is essential for early outbreak warning. Accordingly, substantial efforts have optimized analytical methods for pathogen detection in wastewater^20,21^, and improved the use of WBS data to track and predict regional outbreaks, including hotspot identification using robust datasets^22^, and related analytical approaches^23^. However, viral RNA persistence in wastewater is influenced by wastewater physicochemical properties (e.g., pH, temperature, and organic/inorganic constituents) and environmental factors such as transport/retention time in sewer networks^24,25^. Thus, failing to account for in-sewer decay and related processes can bias epidemiological inference from wastewater RNA measurements (e.g., incidence- or prevalence-based interpretation), potentially underestimating community infection burden^26^.

Several studies evaluated the decay of viable SARS-CoV-2 and SARS-CoV-2 RNA across various water matrices (e.g., wastewater, river water, groundwater, tap water, and seawater)^27–29^ and under diverse environmental conditions (e.g., pH, temperature, and microbial concentrations)^9,28–31^. These studies, among the physicochemical factors, highlighted temperature as a significant factor affecting SARS-CoV-2 RNA reduction^27,28,31^. It is demonstrated that RNA decay was accelerated at 37 ℃ (85% in 1 day) compared to 20 ℃ (60% in 7 days)^28^. It is stated that a higher mean first-order decay rate constant (*k*) was observed at 37 ℃ (0.286 d^− 1^) than that at 4 ℃ (0.084 d^− 1^), 15 ℃ (0.114 d^− 1^), and 25 ℃ (0.183 d^− 1^), respectively^31^. Additionally, the decay rate of SARS-CoV-2 RNA is accelerated by microorganisms in wastewater and by the presence of biofilms in sewer systems^32^, indicating the possible impact of microbial concentration on viral RNA degradation. The decay rate of RNA is higher in raw wastewater than in pasteurized/autoclaved (or disinfected) wastewater after filtration^31,33^. The RNA concentration can be reduced by the adsorption of RNA on mature sewer biofilms^30^, as well as by the diverse physicochemical and biological characteristics of wastewater. The travel time in sewer systems can further influence decay due to continuous exposure to biological and chemical factors^34,35^. For instance, coronavirus concentration decreased by 5.2% to 6% after 2 h of travel in sewers at 20 ℃ to 30 ℃)^35^. While most studies have investigated the impact of environmental and biological factors on virus decay using batch tests or laboratory-scale bioreactors, recent research has increasingly expanded to more complex, real-world systems, addressing this research gap^32,36^.

However, static batch experiments cannot reproduce key sewer transport features—such as travel distance, hydraulic residence time, and evolving biofilms—and they also overlook variable flow conditions that shape in-sewer attenuation. To capture these sewer-relevant processes, we employed a lab-scale sewer pipeline simulator operated under controlled HRT to impose defined travel distances under continuous circulation. Using both batch tests and the simulator, we quantified how pH, temperature, microbial concentration, and sewer travel time influence HCoV-NL63 RNA decay. This integrated design enables more realistic interpretation of wastewater RNA signals and supports WBS applications across diverse sewer system contexts.

## Results

### Influence of physicochemical factors on viral decay rates in wastewater

To identify the key environmental factors influencing the decay rate of the HCoV-NL63 in wastewater, batch tests were conducted under various conditions: pH (2, 5, 7, and 8), temperature (20 ℃, 30 ℃), microbial concentrations (Raw wastewater, 10^− 1^, 10^− 2^, 10^− 3^ diluted wastewater), and SS (216, 133, 74 mg/L). The decay rate of the HCoV-NL63 was significantly highest in wastewater under the combined condition of pH 7 and 30 ℃ (Table 1). Notably, during the incubation period between day 2 and day 5, rapid degradation of viral gene concentrations was observed under the condition of pH 7 at 30 °C. (viral decay constant, *k*, 2.21 d^− 1^; 6.64(± 0.39) log reduction) than other pH conditions (pH 2, 5, 8; *p* < 0.0001) and at 20 °C (*p* < 0.001) (Table 1, Supplementary Fig. S1C). This finding suggests that pH 7 and 30 °C in wastewater have a considerable impact on the decay rate of the HCoV-NL63 (Table 1, Supplementary Fig. S1C).

**Table 1:** Viral decay rate in wastewater influent according to pH and temperature during a 25-day incubation (*n* = 3). Values are mean ± SD; *k* and R² were estimated from the linear phase of ln(C_t_/C_0_) versus time.

pH	Temperature (℃)	NL63 virus decay rate		95% CI of *k*	^a^R^2^
		ln (C_t_/C_0_)	*k*(d^− 1^)		
2	20	-2.29 (± 0.56)	0.76	0.38–1.10	0.98
	30	-3.85 (± 0.30)	0.77	0.60–0.94	0.97
5	20	-2.13 (± 0.31)	0.71	0.23–1.10	0.95
	30	-5.22 (± 0.57)	1.74	0.64–2.72	0.96
7	20	-3.77 (± 1.28)	0.94	0.40–1.25	0.93
	30	-6.64 (± 0.39)	2.21	0.93–3.47	0.97
8	20	-2.98 (± 0.50)	0.75	0.46–0.98	0.96
	30	-5.27 (± 0.31)	1.76	0.61–2.80	0.96

^a^ R^2^ was obtained from linear regression of ln(C_t_/C_0_) versus time over the fitted period used to estimate *k*.

Comparatively, HCoV-NL63 RNA (N gene) decayed faster in wastewater influent than in tap water across all pH conditions, with generally higher *k* values and a clearer increase at 30 °C. Among the tested conditions (pH 2, 5, 7, 8; 20 °C and 30 °C), the highest decay rate was observed at pH 7 and 30 °C (*k* = 2.21 d⁻¹). In contrast, at pH 2, decay constants were similar at 20 °C and 30 °C (Table 1 and Supplementary Table/Fig. S1), indicating no evident additional temperature-driven increase under strongly acidic conditions. Overall, these results suggest that temperature effects are matrix- and pH-dependent rather than strictly monotonic, potentially reflecting changes in virus–particle partitioning and/or dominance of pH-driven inactivation pathways within experimental variability^37–39^.

### Biological factors in wastewater have a substantial impact on viral decay rates

**Fig. 1:**
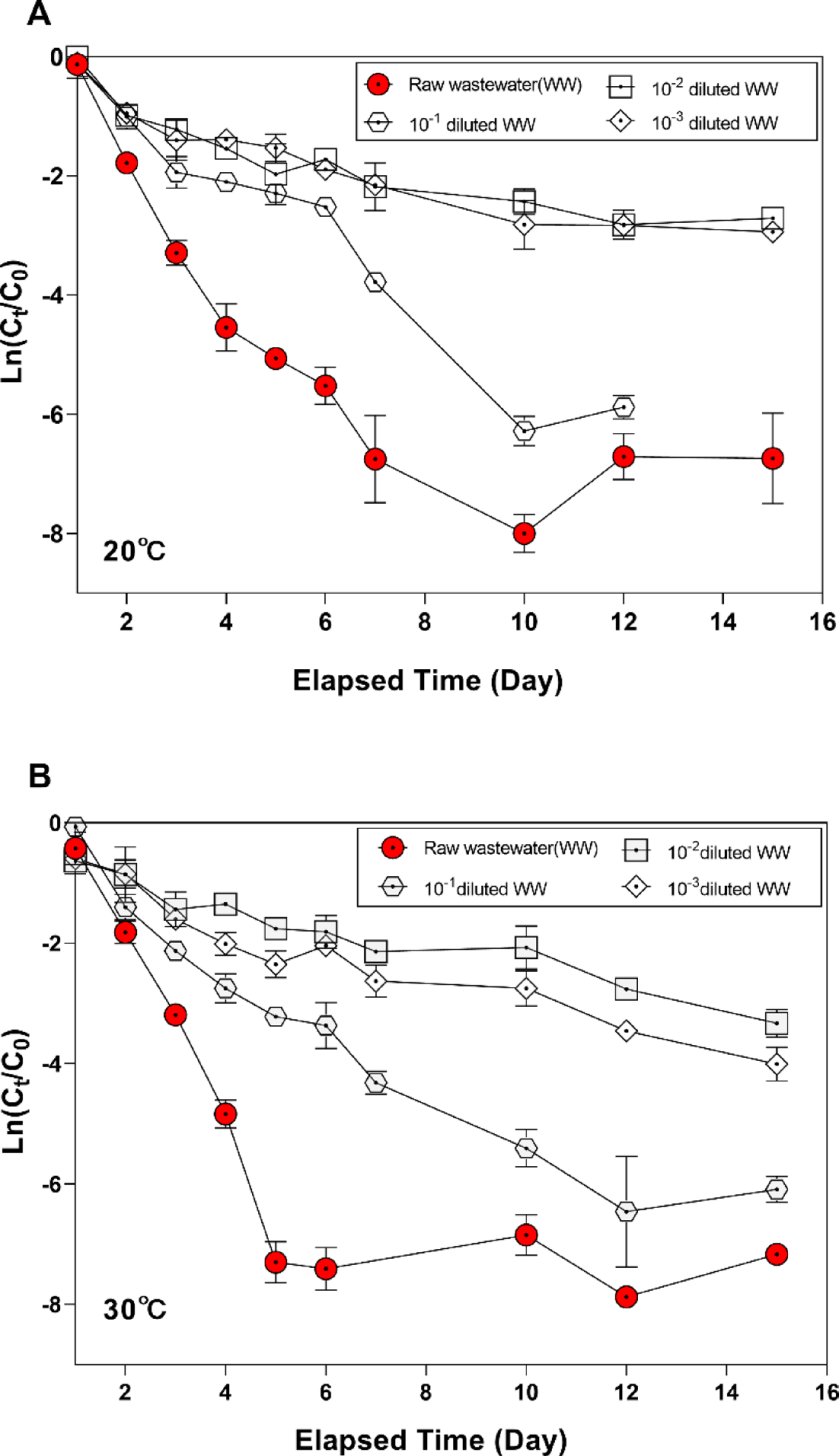
Evaluation of HCoV-NL63 decay rates as a function of microbial concentration in wastewater at pH 7 during a 15-day experiment (*n* = 3 independent replicates). Microbial concentrations were reduced by serial dilution of raw wastewater (10^−1^, 10^−2^, 10^−3^, and 10^−4^). Panels: (**A**) 20 °C; (**B**) 30 °C.

To assess the impact of microbial activities on the decay rate of HCoV-NL63, the batch tests were employed by using wastewater influent with various microbial concentrations at 20 ℃ and 30 ℃, respectively. The results show that the higher the microbial concentration, the faster the viral decay rate (Fig. 1). Specifically, in raw wastewater with a microbial concentration of 7.9 × 10⁶ CFU/mL, the ln(C_t_/C_0_) value reached − 6.90 (± 0.34) from day 1 to day 5 (*p* < 0.0001), and these values gradually decreased as the microbial concentration decreased at both 20 °C and 30 °C (Supplementary Table S1). Although similar patterns were observed at both 20 °C and 30 °C, the decay rate increased more rapidly at 30 °C than at 20 °C. This result indicates that higher microbial concentrations are associated with faster viral RNA decay under the tested conditions. Additionally, the viral decay rate increased earlier at 30 °C than at 20 °C (Fig. 1). These findings demonstrate that the activity of microorganisms, influenced by temperature, affects viral decay rates.

### Influence of suspended solids on viral decay rates in wastewater

**Fig. 2:**
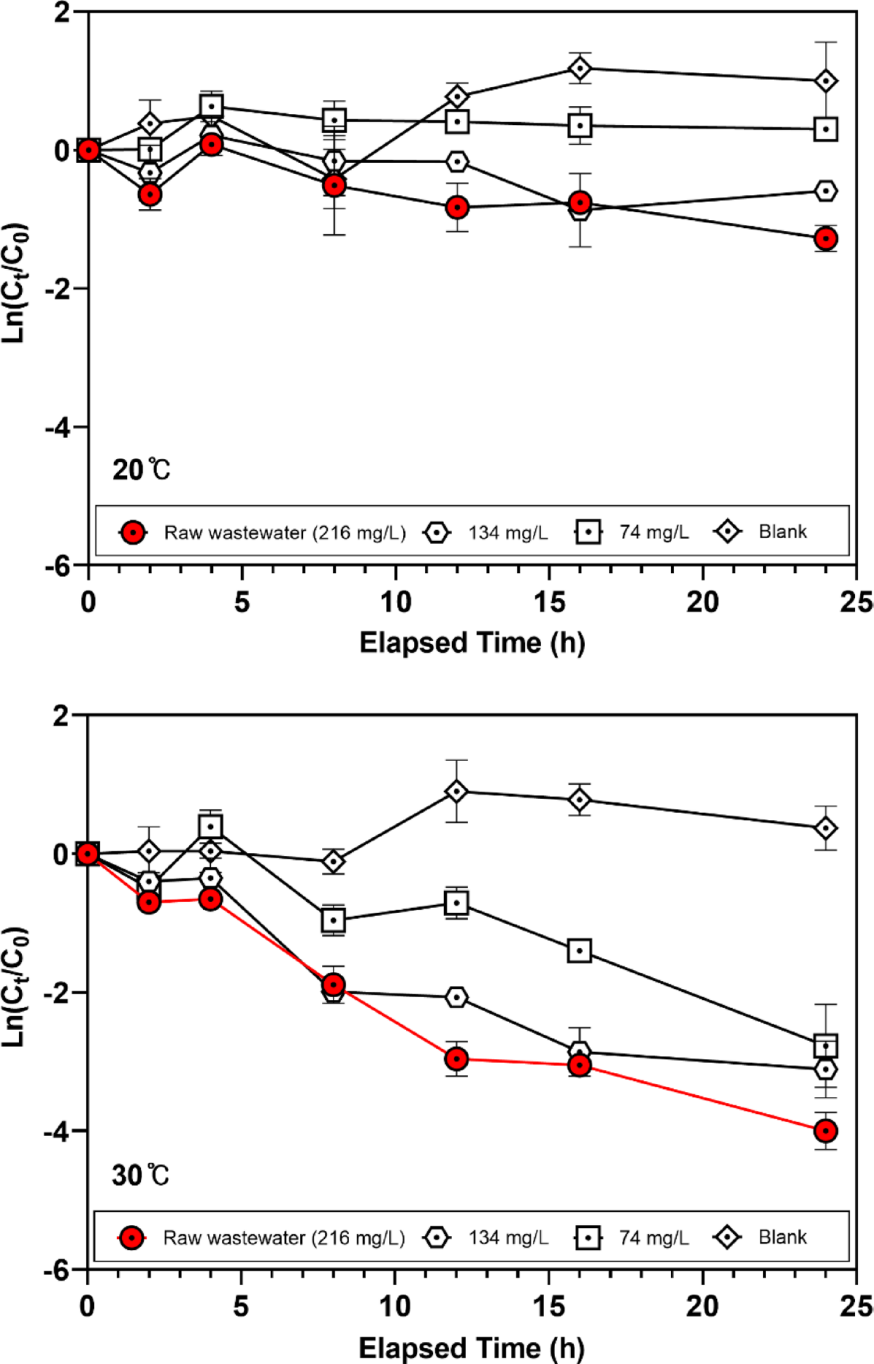
Decay of HCoV-NL63 as a function of suspended solids (SS) concentration during a 24-h experiment (*n* = 3 independent replicates) under (**A**) 20 °C and (**B**) 30 °C. Wastewater samples were adjusted to SS concentrations of 216, 133, and 74 mg L^−1^. Filtered wastewater (0.22 μm membrane) served as the blank control (SS = 5 mg L^−1^).

The concentration of SS in wastewater was demonstrated to significantly influence the decay rate of viruses, including HCoV-NL63^40^. This study aims to evaluate the effect of varying SS concentrations on the decay rate of HCoV-NL63 under controlled batch conditions at different temperatures over a 24-hour period. Consistent with the microbial effect observed above, at 30 °C in raw wastewater (SS = 216 mg/L), the decay rate constant reached *k* = 4.0 d⁻¹ (*p* < 0.0001), which was higher than that observed under diluted wastewater conditions. (Supplementary Table S2). The data also revealed clear temperature dependence, with viral decay rates increasing more rapidly at 30 °C than at 20 °C (Fig. 2). These findings underscore the critical role of SS in enhancing the viral decay rates, with temperature serving as a synergistic factor. The results of this study suggest that higher concentrations of SS are correlated with faster decay of HCoV-NL63 in wastewater. Importantly, the net effect of solids on wastewater-based surveillance is not unidirectional^41^. Many studies have shown that viral RNA can partition to particulate matter/settled solids, which may increase measured analyte concentrations and improve detectability in WBS^40,42^. However, solids can also introduce practical trade-offs, including increased sample heterogeneity, adsorption-associated losses during processing, and co-extraction of PCR inhibitors^21,41^. Therefore, our SS-related findings should be interpreted as context-dependent, reflecting a balance between potential enrichment via partitioning and concurrent losses and analytical challenges associated with solids-rich matrices^24,40^. This finding is consistent with the influence of microbial factors observed in similar batch tests. This correlation reinforces the hypothesis that both SS and elevated temperatures are key drivers of viral decay in wastewater.

### Integrated impact of microbes and suspended solids on virus decay rate

**Fig. 3:**
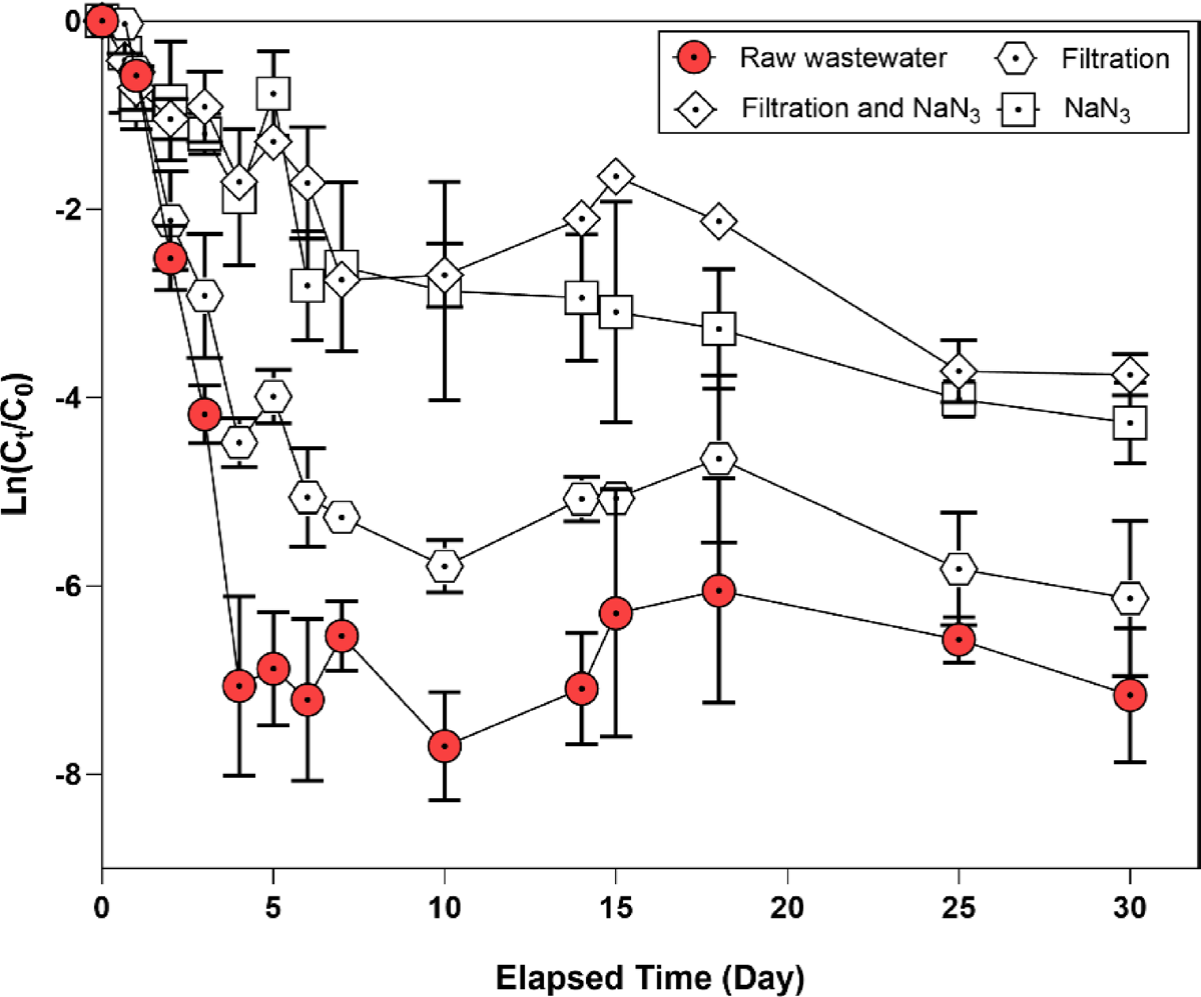
Batch test for HCoV-NL63 decay in wastewater containing both suspended solids (SS) and microbes. (A) Decay rates of HCoV-NL63 in different matrices during a 30-day experiment at 30 °C (*n* = 3 independent replicates): raw wastewater, GF/C-filtered wastewater, GF/C-filtered wastewater treated with 0.1% NaN_3_, and raw wastewater treated with 0.1% NaN_3_.

To elucidate the relative impact of microbial concentration and SS on the decay rate of HCoV-NL63, batch tests were performed using four different wastewater conditions. The results indicate that the multifactorial impacts of the microbial concentrations and SS on the decay rate of HCoV-NL63 were greater than those of the SS and microbial concentrations, respectively (Fig. 3). Specifically, HCoV-NL63 decayed the fastest in raw wastewater, followed by filtered wastewater. This demonstrates the influence of microbial activity and SS concentration on viral decay. The ln(C_t_/C_0_) value for raw wastewater was − 7.06 (± 0.95, *p* < 0.0001), indicating the fastest decay among the tested conditions. In contrast, the value for filtered and 0.1% NaN_3_-treated wastewater was − 1.04 (± 0.22, *p* = 0.0051), representing the slowest viral decay. (Supplementary Table S3). This demonstrates that the viral decay rate was markedly reduced following 0.1% NaN_3_ treatment. These results indicate that microbial activity has a stronger impact on viral decay, while SS concentration also plays a role.

Further analysis of raw and 0.1% NaN_3_-treated wastewater highlights the significant impact of microbial activity on viral decay, compared to the effect of SS (Fig. 3). From days 2 to 10, the ln(C_t_/C_0_) values were − 1.68 (± 0.34, *p* < 0.005) in filtered and 0.1% NaN_3_-treated wastewater, and − 2.13 (± 1.16, *p* < 0.005) in 0.1% NaN_3_-treated wastewater, respectively. The filtration step reduces both microbial and SS concentrations, whereas 0.1% NaN_3_ specifically inactivates microbial activity. These results suggest that the influence of SS is relatively weak compared to that of microbial concentration.

### Viral decay in laboratory-scale sewer pipeline simulator

**Fig. 4:**
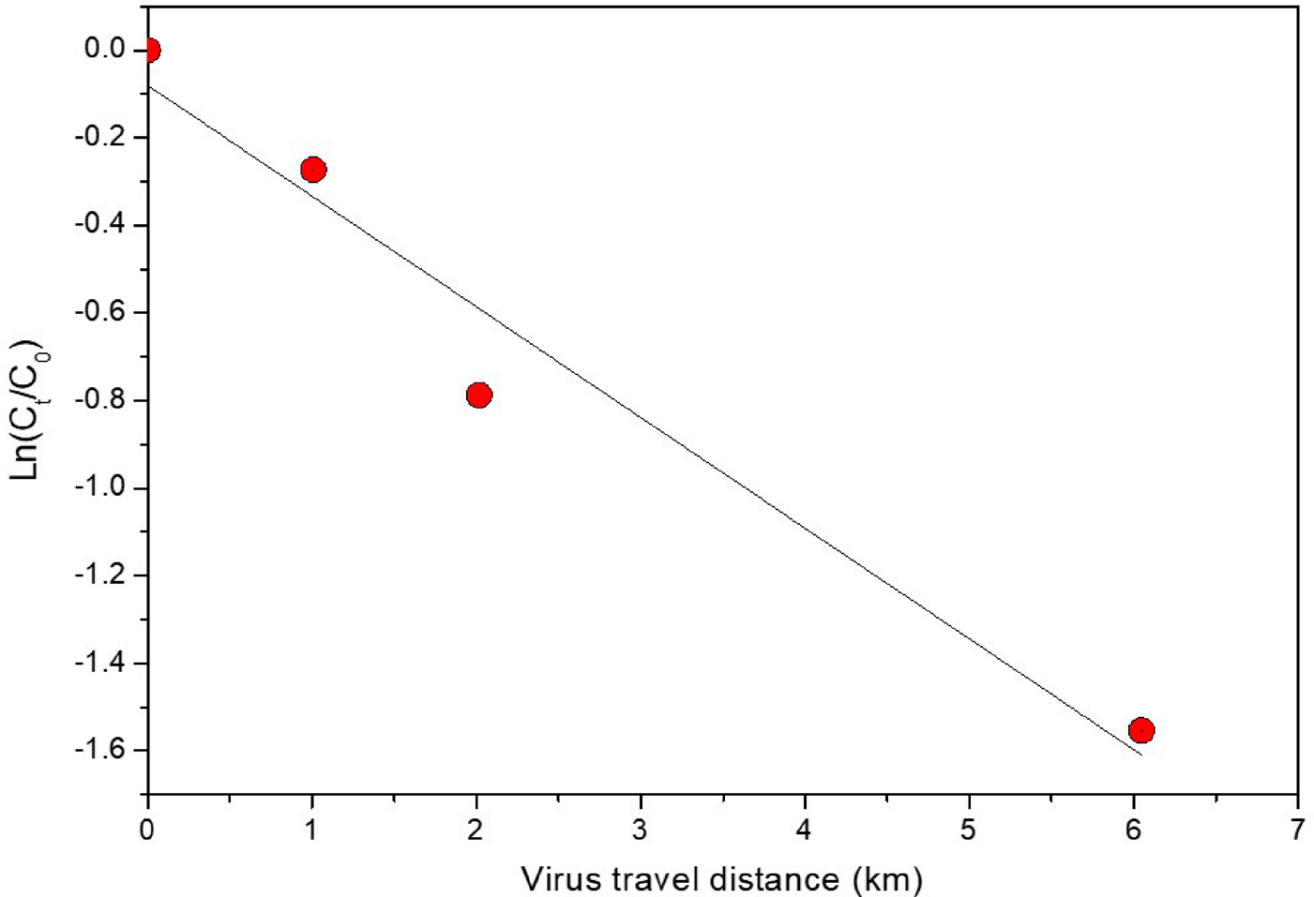
Degradation of HCoV-NL63 gene concentrations as a function of virus travel distance in the sewer pipe simulation system during the tap-water (2-day) and wastewater (7-day) operations (duplicate samples, *n* = 2).

When the dechlorinated tap water was applied as the influent, the coronavirus concentration decreased by 51.7% within 2 days (from initial concentration of 9.1 × 10^7^ GC L⁻¹ to 4.7 × 10^7^ GC L⁻¹), while when sewage was applied, it decreased by 76.1% within 2 days (from 5.07 × 10^6^ GC L⁻¹ to 3.9 × 10^6^ GC L⁻¹). As a result of data analysis based on the first-order decay model, the decay rate constant (*k*) values ​​in tap water and sewage were 0.28 and 0.52 d^− 1^, respectively, showing that the virus decay rate is faster in sewage (Table 2). The virus concentration and decay rate according to the virus movement distance are shown in Fig. 4. According to the first-order decay model, the *k* and *T*_*90*_ values ​​according to the travel distance were 0.25 km^− 1^ and 9.2 km, respectively. Specifically, if a biofilm is not formed in the sewer pipe and the virus moves 9.2 km through the sewer pipe, the virus concentration decreases by 1 log. However, because various types of organic and inorganic sediments and biofilms are formed in sewage pipes, it is believed that the actual *T*_*90*_ value will be much smaller than that derived in this study.

**Table 2:** Decay rate of HCoV-NL63 derived from the sewer pipe simulation process during continuous operation with dechlorinated tap water (2 days) and wastewater (7 days) (duplicate samples, *n* = 2).

Decay of NL63	Tap water	Wastewater	
		Stage I	Stage II
*k* (d^−1^)	0.28	0.52	1.20
*t*_*90*_ (d)	8.2	4.4	1.9

**Fig. 5:**
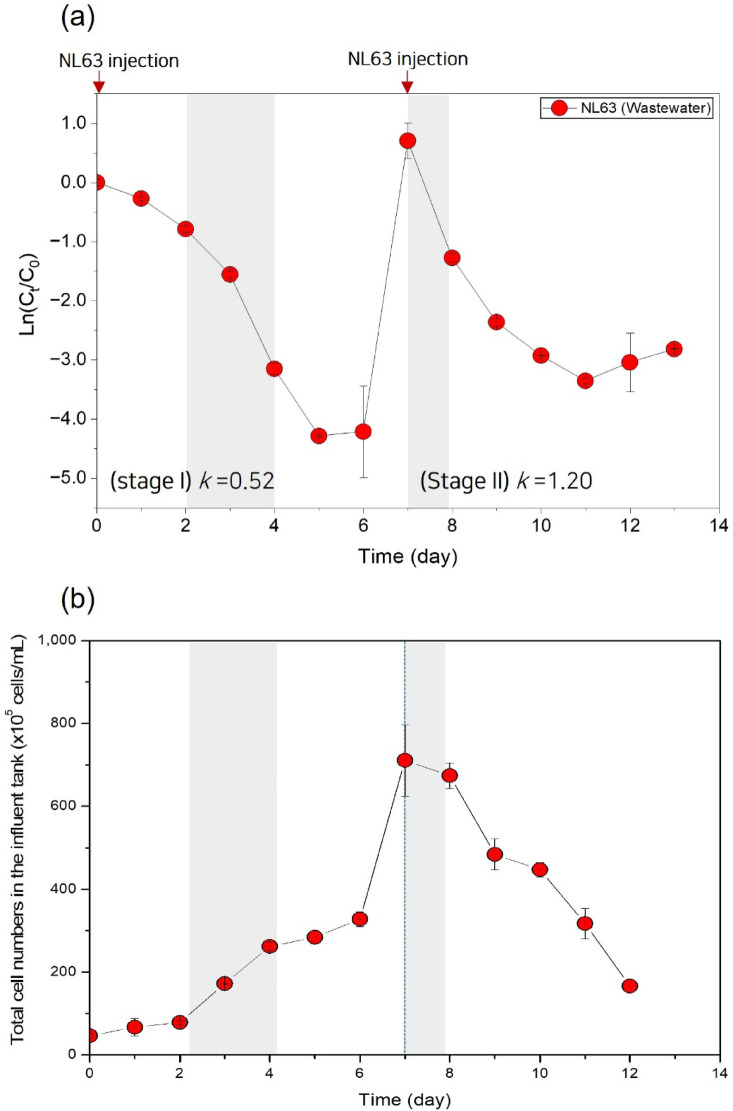
(**a**) Decay of HCoV-NL63 in sewage during the sewer pipeline simulation process and (**b**) changes in the total number of microorganisms in the influent tank during the same operation (dechlorinated tap water: 2 days; wastewater: 7 days; duplicate samples, *n* = 2).

Under constant temperature conditions at 25 ℃, the *k* values ​​of coronavirus NL63 in tap water and sewage were 0.28 and 0.52 d^−1^, respectively, and the time for 90% of the virus to decrease (*T*_*90*_) was 8.2 and 4.4 days, respectively, based on the 2-day results. Because in-sewer temperatures vary by season, geography, and hydraulic conditions, our 25 °C simulator results should be interpreted as controlled mechanistic evidence rather than a direct representation of all field scenarios^43,44^. After the second NL63 virus injection (stage II), compared to that of the first virus injection period (stage I), the viral decay rate was more than doubled to 1.20 d^−1^, and the time for *T*_*90*_ to decrease to 1.9 days was also confirmed to be shorter (Table 2). Figure 5 shows the changes in the total number of microorganisms in the influent tanks. In stage I, the number of microorganisms increased rapidly when the virus concentration in the sewage decreased (2–4 days), and during stage II, the total microorganism abundance showed a mild but steady decrease over time. These observations suggest that viral RNA reduction during continuous operation may be associated with microbial dynamics in sewage.

The reported first-order decay coefficients (*k*) for coronaviruses in wastewater vary across studies depending on experimental conditions, ranging from 0.15 to 1.75 d⁻¹ (Table 3)^28,31,35,45^. The *k* value estimated in this study (0.52 d⁻¹) falls within this range^35^. As described above, the decay rate of coronaviruses in sewage was evaluated on a laboratory scale by analyzing the simple mixing reaction of sewage and viruses^28,31,35^, and in sewer systems. In this study, we analyzed the viral decay rate according to the characteristics of influent water (tap water and sewage) using a sewer pipe simulation process, and the concentration (activity) of microorganisms in the influent water was identified as an environmental influencing factor for the increase in the viral decay rate.

**Table 3:** Comparison of coronavirus decay rates with published studies.

Water matrix	Experimental set-up	Reaction time (day)	Spiked virus	Target gene	Decay rate, k (d^− 1^)	Half-life, t _1/2_ (day)	t _90_ (day)	Reference
Wastewater	Batch test	33	Gamma-irradiated SARS-CoV-2	N1	0.15	4.78	15.88	^31^
Wastewater	Batch test	1	^a^None	N1, N2	1.75	0.40	1.31	^45^
Wastewater	Batch test	6	^a^None	N1	0.84	0.83	2.74	^35^
Wastewater	Batch test	7	SARS-CoV-2 nCoV-WA1-2020	E	0.67	0.99	3.30	^28^
Wastewater	Lab-scale sewer pipeline simulator	5	HCoV-NL63	N	0.52	2.04	4.40	This study

^a^ Detection of intrinsic SARS-CoV-2 viral gene concentrations in wastewater.

## Discussion

This study investigates the environmental factors influencing viral RNA degradation in a sewer system using batch tests and a laboratory-scale sewer pipeline simulator. The findings provide insights into how specific conditions—such as pH, temperature, suspended solids (SS), and microbial concentration—affect the decay rate of HCoV-NL63 RNA. Overall, the batch tests demonstrate that viral decay is most pronounced under conditions favorable for microbial activity and growth, highlighting the interplay between environmental and biological processes.

We further evaluated pH and temperature effects. For real-world wastewater surveillance, interpretation focuses on near-neutral conditions (pH 5–8), whereas the highly acidic condition (pH 2) is included only as a boundary stress case. At 30 °C, HCoV-NL63 RNA decreased by 5.22, 6.64, and 5.27 log at pH 5, 7, and 8, respectively (Table 1). The rapid decay at pH 5–8, typical of sewage, is likely associated with enhanced microbial activity^41^, including enzyme and metabolite production that promotes viral particle degradation. Interactions with wastewater organic matter may further facilitate decay, supporting the interpretation that biological processes are the primary drivers of viral RNA loss in sewer systems^46^.

Beyond pH and temperature, biological factors (microbial activity) and physical factors such as suspended solids (SS) also influence viral decay. The pH range tested (2–8) was selected to examine decay across a broad spectrum rather than to represent typical wastewater conditions. Decay was most pronounced at pH 7, indicating that near-neutral pH can be associated with rapid RNA loss under our experimental conditions. Although wastewater is generally near neutral, evaluating this wider pH range provides insight into potential persistence or loss under extreme pH conditions^4,47^.

Because pH 2 is not representative of typical municipal wastewater, we treat it as a boundary-case sensitivity test and restrict real-world interpretation to near-neutral pH conditions. Nevertheless, transient mildly acidic conditions may occur in practice due to episodic inputs (e.g., industrial/chemical discharge) or localized sewer microenvironments, supporting the inclusion of pH 5 as a lower-end plausible condition. At pH 2 and 20 °C, viral RNA showed a relatively small reduction (2–3 log) compared with near-neutral pH. This strongly acidic pH and lower temperature are suboptimal for microbial activity, likely reducing enzyme production and biological contributions to viral degradation^38^. In addition, capsid stability under acidic conditions may help preserve viral particle integrity, contributing to slower decay. Accordingly, the decay coefficient derived from the tap-water run is presented as a short-window baseline and is interpreted primarily over the time window common to both tap water (48 h) and wastewater experiments. Overall, these results suggest that although microbial activity is a critical driver of viral degradation under typical wastewater conditions, extreme pH can inhibit these processes and potentially prolong viral persistence^48^.

Seasonal and anthropogenic factors can also shift wastewater conditions beyond pH and temperature. For example, pollutants and rainfall intrusion can alter wastewater properties; wastewater temperatures of 27–29 °C may decrease to ~ 25 °C after rainwater intrusion^43^. Seasonal pH differences have also been reported, with median values of 7.36 in summer and 6.79 and 6.62 in autumn and winter, respectively^44^. Although these changes are relatively modest, they may still affect microbial activity and influence measured viral RNA concentrations in sewers or at WWTPs.

Beyond physicochemical factors, biological components—especially microbial activity and suspended solids (SS)—are major drivers of viral decay. Higher microbial concentrations can accelerate decay by enhancing predation, enzymatic activity, and biofilm formation. For example, decay has been reported to be up to threefold higher in eutrophic than in oligotrophic waters^49^, and membrane bioreactors show substantial virus removal via predation and enzymatic breakdown^50^. Enveloped viruses also decay faster in untreated wastewater than in pasteurized wastewater, underscoring the role of active microbial communities^24^. In addition, biofilms can trap and inactivate viruses^51^, and decay rates for coronaviruses are consistently higher in wastewater than in other aquatic environments, suggesting that microbially derived processes contribute to viral inactivation^52^.

Building on these biological effects, microbial activity exerted a stronger influence on viral decay than physicochemical attenuation associated with suspended solids (SS) (Fig. 3). After GF/C filtration, the decay rate constant was *k* = 1.12 d⁻¹, only slightly lower than in raw wastewater, whereas inhibiting microbial activity with NaN_3_ reduced *k* to 0.47 d⁻¹ (Supplementary Table S3). Because GF/C filtration can decrease microbial abundance as well as SS, this condition should not be interpreted as a solids-only treatment, and mechanistic attribution should be made cautiously when comparing filtration and NaN_3_ inhibition^53,54^. Overall, physicochemical and microbiological attenuation during wastewater transport can bias measured viral RNA signals, highlighting the need to account for decay when interpreting wastewater surveillance data^28,36,55^.

Microbial communities can accelerate viral RNA loss in wastewater through multiple mechanisms, including protozoan grazing/adsorption and enzymatic degradation of virions and released RNA^56,57^. Wastewater-associated surfactants/detergents and quaternary ammonium compounds may further disrupt lipid envelopes, and extracellular nucleases may hasten RNA breakdown once particles are compromised^58–60^. Because we did not chemically profile these constituents, we discuss them as plausible co-varying contributors rather than definitive drivers. Overall, comparisons between untreated and filtered/autoclaved matrices support microbial activity as the dominant driver of decay in our system^24,32,33^. We also note that influent holding time and cold storage can modulate microbial activity; extended storage, when unavoidable, may add uncertainty to microbiota-based interpretation^31,36^. Finally, because the microbial-concentration experiment relied on serial dilution, dissolved chemicals likely co-varied with microbial abundance; thus, the observed trend should not be interpreted as evidence that microbial concentration alone is the sole causal driver of RNA decay.

**Fig. 6:**
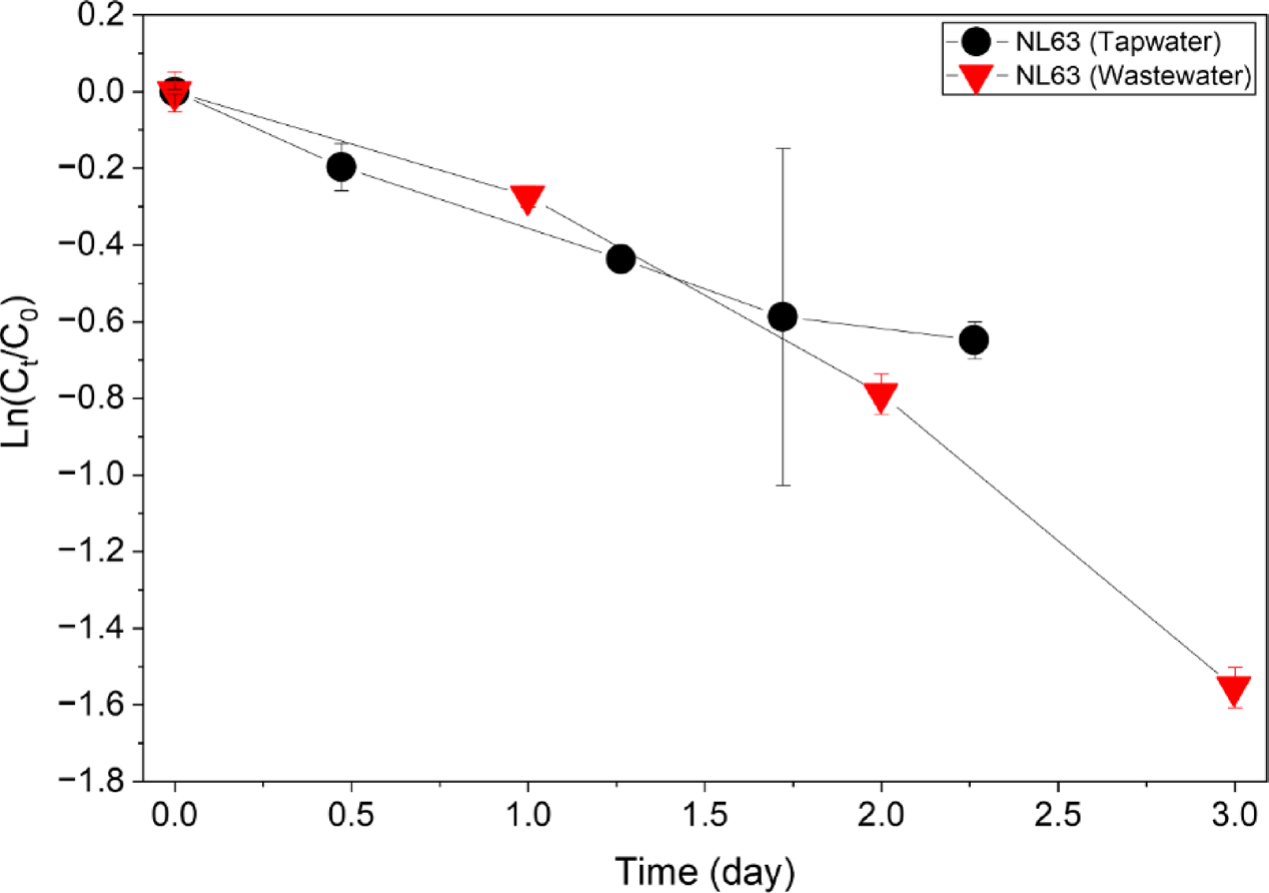
Virus decay during the sewer pipe simulation process using dechlorinated tap water (2-day operation) and wastewater (7-day operation) as influent (duplicate samples, *n* = 2).

Consistent with prior decay studies comparing wastewater with cleaner water matrices, we observed faster viral RNA decay in untreated wastewater than in tap water (Fig. 6)^9,31,61^, suggesting that wastewater-specific constituents and biological activity contribute to enhanced decay. In lab-scale sewer pipeline simulator tests, viral decay increased with travel distance and the presence of sewer biofilms (Fig. 4), and this trend co-occurred with increasing bacterial concentrations^39^. Previous studies similarly show that temperature and other environmental conditions affect the decay rate constant (*k*) of SARS-CoV-2 in untreated wastewater^31,62^, highlighting that water matrix properties, microbial activity, and biofilms jointly shape coronavirus persistence^39,63^. These observations underscore the need to consider in-sewer attenuation processes when interpreting WBS signals and designing treatment strategies.

In real sewer networks, intermittent or continuous wastewater inputs promote biofilm development on pipe surfaces. Biofilms can modulate viral persistence through adsorption/partitioning and microbially mediated processes, potentially contributing to in-sewer attenuation. In our simulator, we operated the tubing under light-shielded conditions and observed operational evidence consistent with biofilm accumulation over time; however, we did not include photographic documentation because the images did not provide sufficiently interpretable structural confirmation. Future studies should incorporate quantitative biofilm measurements (e.g., biomass, thickness, or surface-associated nucleic acid) to directly link biofilm development with viral RNA stability and transport.

In-sewer decay effects on SARS-CoV-2 WBS are context dependent, varying with temperature and wastewater composition^35,64,65^. This is most consequential when RNA levels approach detection limits during endemic/low-incidence periods^66–68^. Because influent concentrations span ~ 10⁰–10² to ≥ 10³ gene copies mL⁻¹^13,14,64^, WBS requires sensitive, target-validated workflows whose performance can be matrix dependent^20,69,70^. Decay- and transport-aware interpretation can further reduce underestimation^34,71,72^. The present study employs HCoV-NL63 as a controlled and safer experimental model to investigate how wastewater conditions and analytical workflows influence measured RNA decay signals; however, we explicitly acknowledge that the response of HCoV-NL63 to specific concentration procedures, and its comparability to SARS-CoV-2, remains to be established and is beyond the scope of this study.

Environmental and biological factors in wastewater can modulate viral decay rates and potentially bias wastewater-based estimates of community infection burden. Wastewater RNA should therefore be interpreted as an aggregate signal shaped by both shedding and in-sewer processes (e.g., dilution, adsorption/partitioning to solids and biofilms, and microbially mediated decay), rather than as a direct proxy for clinical incidence or prevalence. In our data, decay rates from spike-in experiments were higher than those inferred from intrinsic signals (Table 3)^24^. This discrepancy likely reflects differences in the physicochemical state of the target (freshly spiked material vs. matrix-associated endogenous RNA) and its partitioning/retention by suspended solids and biofilms, which can prolong detectability of intrinsic signals^39,73^. Therefore, decay constants should be interpreted based on whether they derive from spike-in or intrinsic measurements, rather than treated as directly interchangeable metrics.

If in-sewer decay is not accounted for, measured RNA concentrations may underestimate the true community infection burden, particularly in catchments with longer conveyance times and warmer temperatures. This is important because key determinants—temperature, pH, suspended-solids-associated partitioning/adsorption, microbially mediated decay (e.g., extracellular nucleases), and biofilm- and conveyance-time effects—can vary across locations and system types, thereby influencing viral persistence and detectability in wastewater^24,74–76^. Among these factors, temperature is a critical driver of RNA stability and should be considered when interpreting wastewater surveillance data; otherwise, viral loads may be underestimated or misinterpreted^77^. Further work is needed to clarify how wastewater properties across industrial, agricultural, and residential settings affect the detection and quantification of viruses, including SARS-CoV-2, in WBS.

Building on these considerations, SARS-CoV-2 RNA persistence in wastewater and nearby surface waters has been widely reported, despite the general tendency of enveloped viruses to degrade in natural aquatic environments. For example, limited decay (< 1 log10 reduction) of SARS-CoV-2 RNA was observed in primary settled solids over 10 days at 4, 22, and 37 °C^42^. Another study reported that 51–68% of the initial SARS-CoV-2 RNA remained detectable in sewer biofilms after three cycles of replacement with virus-free wastewater^39^. Long-term monitoring in Brazil also consistently detected SARS-CoV-2 RNA in untreated wastewater over 44 weeks, with concentrations ranging from 2.7 to 7.7 log10 GC L⁻¹^78^.

However, because most wastewater studies quantify SARS-CoV-2 RNA rather than infectious virus, the persistence of infectious SARS-CoV-2 in wastewater and the associated transmission risk remain uncertain^9,28^. RNA detection indicates persistence of genetic material but does not necessarily reflect infectivity; accordingly, RT-qPCR/RT-ddPCR signals should not be interpreted as a direct proxy for infectious virus decay^79,80^. We therefore restrict our conclusions to RNA decay kinetics of spiked HCoV-NL63 under controlled wastewater conditions^81–83^. Future studies should integrate complementary approaches (e.g., capsid-integrity/viability RT-qPCR and cell culture–based assays) to better link molecular signals to infectivity and potential risk^79,80^.

Considering these factors, integrating viral decay kinetics and environmental variability into wastewater surveillance is important to improve the accuracy of pathogen detection and strengthen public health decision-making. Incorporating virus- and matrix-specific decay rates into WBS models can reduce interpretive bias and improve tracking of infectious disease dynamics across systems, thereby supporting more effective public health interventions.

The pipeline simulator extends batch tests by adding continuous circulation and defined travel distance. The shorter tap-water run provides only a short-term low-bioactivity baseline. Limitations include limited replication (*n* = 2–3), spike levels above typical field ranges^13,84^, an open reservoir^36,72^, nonstandard tubing materials^39,85^, and cell-culture virus that may behave differently from solids-associated human shedding^86,87^. We therefore interpret results as apparent decay of spiked HCoV-NL63 RNA and caution against direct field extrapolation^64,88^.

We did not correct measured RNA concentrations for method recovery efficiency^68^. Therefore, the decay constants reported here should be interpreted as apparent decay rates under a fixed workflow, and additional uncertainty could arise if recovery were to vary over time within a condition. Although recovery controls can verify consistent processing, surrogate recovery can vary substantially across matrices and may not represent target losses across all processing steps; therefore, recovery-based correction may introduce additional bias. Accordingly, we report uncorrected concentrations and interpret fitted decay constants as apparent RNA decay derived from within-condition time series processed identically^68^.

Our findings confirm the presence of viral RNA in wastewater as detected by qPCR. As noted above, qPCR/RT-ddPCR measurements cannot distinguish infectious from non-infectious particles^79^; therefore, our interpretation is restricted to RNA decay kinetics under controlled conditions, and infectivity-based assessments (e.g., culture-based assays or capsid-integrity/viability RT-qPCR) remain an important direction for future work.

In this study, HCoV-NL63 was propagated in LLC-MK2 cells following ATCC protocols^89^. While RNA stability was assessed by RT-qPCR, TCID₅₀ assays were performed during virus preparation to verify infectivity potential^90^; because our focus was RNA stability, detailed TCID₅₀ results are not presented. Future studies combining RNA decay and infectivity measurements would provide a more comprehensive understanding of HCoV-NL63 persistence under varying conditions. Using batch tests with a 15-day incubation and a lab-scale sewer pipeline simulator, we found that microbial activity was the dominant factor accelerating viral RNA decay, particularly under conditions favorable for microbial growth at near-neutral pH and elevated temperature.

The “2-day” simulator duration is an observation window, not a typical sewer residence time^91^; conveyance varies with network hydraulics and storage^35,36,72^. With *k* = 0.52 d⁻¹ at 25 °C, attenuation over 6–12 h is small (~ 0.06–0.11 log₁₀) but increases with longer residence times [36]. We included 30 °C as an upper-temperature sensitivity case relevant to warm periods and climates^92,93^.

This approach highlights the lab-scale sewer pipeline simulator as a novel contribution to WBS. By bridging laboratory batch tests and sewer-relevant transport conditions, it provides more realistic insight into viral RNA persistence and attenuation dynamics. Integrating lab-scale sewer pipeline simulator and batch experiments strengthens WBS interpretation and supports public health monitoring across diverse sewer system contexts.

## Methods

### Wastewater and dechlorinated tap water

Wastewater influent and sludge were collected from a WWTP in Sejong, Republic of Korea, with an average inflow of 25,000 m³ d⁻¹. Based on sewer design standards and an assumed per-capita wastewater generation of ~ 300 L d⁻¹, this capacity corresponds to an estimated serviced population of ~ 100,000. Samples (4 L) were transported on ice at 4 °C and typically processed within ~ 24 h (i.e., experiments initiated the next day). When immediate processing was not feasible, samples were stored at 4 °C for up to 7 days (maximum holding time) before experimentation. Tap water was dechlorinated by aeration for ≥ 24 h. Residual free chlorine was measured using a Free Chlorine ISM (HI 96701; Hanna Instruments, Italy) following the manufacturer’s protocol, and no signal was detected^31^.

### Human coronavirus NL63 preparation

Monkey LLC-MK2 kidney epithelial cells (ATCC, CCL-7) were cultured in T-175 flasks (Thermo Fisher Scientific) in Dulbecco’s modified Eagle’s medium (DMEM) supplemented with 5% (v/v) heat-inactivated fetal bovine serum and 1% (v/v) penicillin–streptomycin (HyClone). Cells were maintained at 37 °C with 5% CO₂, seeded at 1.0 × 10⁴ cells cm⁻², and subcultured every 4 days. Human coronavirus NL63 (HCoV-NL63) was obtained from the Laboratory of Virology at Korea University and propagated in LLC-MK2 cells following ATCC protocols. Briefly, cells were grown to > 80% confluency (72–96 h), inoculated with NL63, and incubated until > 80% cytopathic effect (CPE) was observed. The culture was then freeze–thawed (− 80 °C, then thawed) to increase virus yield, transferred to a 50 mL conical tube, aliquoted (1 mL) into sterile 1.7 mL microtubes, and stored at − 80 °C until use. Before evaluating environmental factors, we assessed viral stock RNA stability during − 80 °C storage by extracting RNA immediately after preparation and again after one year. The initial Ct values (10.25 and 10.59) and post-storage values (10.94 and 10.60) were not significantly different (*p* = 0.28), indicating minimal impact on RNA integrity (Table S5). Consistent with prior reports of prolonged stability of viral nucleic acids at subfreezing temperatures^45,94–96^, these results support − 80 °C storage as sufficient for preservation, although additional validation may be needed for longer durations.

### Batch test under varied environmental conditions

#### Impact of pH and temperature

Batch tests were performed under different pH conditions (2, 5, 7, and 8), adjusted using 1 N H₂SO₄ or 1 N NaOH, and temperature conditions (20 and 30 °C) in wastewater or dechlorinated tap water^97^. All pH treatments were prepared from aliquots of the same well-mixed influent wastewater sample (within each experimental run), and only pH was adjusted to the target values. Test conditions were selected to include both wastewater-relevant ranges and boundary (stress-test) conditions to evaluate the sensitivity of HCoV-NL63 RNA decay to key environmental drivers. Specifically, pH 5–8 was used to reflect near-neutral wastewater conditions, whereas pH 2 was included only as an extreme boundary (stress-test) condition and is not intended to represent typical municipal wastewater; thus, real-world implications are interpreted primarily for the near-neutral range (pH 7–8), with pH 5 treated as mildly acidic. Although strongly acidic conditions are not typical of domestic sewage, mildly acidic episodes (e.g., around pH 5–6) can arise transiently in certain catchments influenced by industrial/chemical inputs or episodic acidic discharges, and potentially within localized sewer microenvironments. Accordingly, pH 5 was included as a plausible lower-end scenario rather than a typical municipal condition, while pH 2 was retained only to bracket sensitivity under extreme acidity. In general, reported sewer/domestic wastewater pH is near-neutral and commonly varies within approximately 6.2–8.5, depending on catchment characteristics and diurnal dynamics^98^. Temperatures (20 and 30 °C) were chosen to represent moderate and warm sewer/wastewater conditions relevant to coronavirus RNA decay. Wastewater temperature varies substantially by climate and season; for example, reported annual values can span roughly 3–27 °C, with warmer-season conditions approaching the upper 20s^99^. Suspended solids (SS) levels were anchored to the measured influent SS concentration and then reduced by controlled dilution/filtration to assess matrix-dependent effects on decay kinetics. Each test was performed in a 15 mL conical tube with a total working volume of 5 mL, with two independent replicates. HCoV-NL63 (5 µL) was spiked into wastewater and dechlorinated tap water to a final concentration of 1.45 × 10⁸ genome copies (GC) L⁻¹ and mixed thoroughly. Over 30 days, 200 µL aliquots were collected 16 times (each in triplicate) and immediately lysed with 600 µL TRIzol^®^ reagent (Invitrogen, MA, USA). Lysates were stored at − 80 °C for up to one month prior to analysis. All samples were processed, and RNA was extracted within one week of collection.

#### Impact of suspended solids

The concentration of suspended solids (SS) was measured according to the Standard Methods for the Examination of Water and Wastewater (23rd edition)^97^. Influent wastewater initially contained 216.0 mg L⁻¹ SS. This influent SS concentration is within commonly reported ranges for untreated domestic wastewater (often on the order of ~ 100–350 mg L⁻¹), supporting the field relevance of the baseline matrix^100^. To prepare lower-SS conditions, raw wastewater was serially diluted 1:1 at each step using 0.22 μm–filtered influent, yielding final SS concentrations of 133.0 and 74.0 mg L⁻¹. Because 0.22 μm filtration can also remove microbial cells, the filtered influent used for dilution/blank preparation was treated as a low-SS matrix that may also have reduced microbial abundance, rather than a solids-only control; this potential confounding was considered when interpreting SS effects. Each diluted condition was prepared at a total volume of 500 mL, and SS was remeasured after dilution to confirm target values. A blank was prepared by filtering raw wastewater through a 0.22 μm filter, which reduced SS to 5 mg L⁻¹ and minimized suspended-solids effects. Each condition was aliquoted (40 mL), spiked with 40 µL of NL63 stock (final concentration ~ 3.03 × 10¹⁰ GC L⁻¹), and vortexed for 30 s. Samples (200 µL) were collected in triplicate at 0, 2, 4, 8, 12, 16, and 24 h and immediately lysed with 600 µL TRIzol^®^ reagent. Lysates were stored at − 80 °C until analysis, and RNA was extracted within one week of collection.

#### Impact of microbial cell concentrations

400 mL of wastewater was subjected to a Labogene 1580R Swing centrifuge, (Labogene, Korea) at 11.8 × g for 5 min at 4 °C to remove large particles. The post-centrifugation liquid phase was collected and designated as clarified wastewater; it was then 10-fold serially diluted with 1×PBS to a final dilution of 1:1000, and each dilution was adjusted to a total volume of 10 mL. Then, 100 µL of each dilution was spread on R2A agar plates (Becton, Dickinson and Company, Franklin Lakes, NJ, USA), and incubated at 37 °C. After at least 15 h, CFU/L unit was calculated by counting colonies in plates. The resulting supernatant contained 7.9 × 10^9^ CFU/L. Serial dilutions from 10^− 1^ to 10^− 4^ were similarly prepared for further experiments. The 499.5 mL of prepared diluted wastewater samples were mixed with 500 µL of HCoV-NL63 (3.03 × 10^10^ GC L⁻¹) and incubated at 20 ℃ or 30 ℃ for 15 days. During the incubation period, 200 µL of samples were taken(*n* = 3), and 600 µL of Trizol™ reagent was added immediately at 0, 2, 4, 8, 16, and 24 h, and subsequently 2, 3, 4, 5, 6, 7, 10, 12, and 15 days. The samples were stored at − 80 ℃ until further RNA extraction. All samples were processed, and RNA was extracted within one week of collection.

#### Impact of SS and microbes

SS concentration was determined as previously described^97^. Four types of wastewater samples were prepared to evaluate which of the two variables—suspended solids (SS) or microbial communities—exerts a greater influence on viral decay rates by (i) filtration with a GF/C filter, which reduces both SS and microbial concentration, (ii) filtration (GF/C filter) followed by the addition of 0.1% NaN_3_ and incubated 3 h to suppress microbial activity, and (iii) addition of 0.1% NaN_3_ and incubated 3 h without filtration, allowing SS to remain while inhibiting microbial activity^54,101^. The pre-treated wastewater samples were incubated at 30 ℃ for 3 h and adjusted to pH 7. All samples (40 mL) were aliquoted, and 40 µL of NL63 virus (3.03 × 10^10^ GC L⁻¹) was injected into the samples. A quantity of 200 µL was taken in triplicate for each sample and immediately at 0, 2, 4, 8, 16, and 24 h, and subsequently 2, 3, 4, 5, 6, 7, 10, 14, 15, 18, 25, and 30 days, added with 600 µL of Trizol^®^ reagent. The samples were stored at − 80 ℃ until further RNA extraction. All samples were processed, and RNA was extracted within one week of collection.

### Lab-scale sewer system set-up

**Fig. 7:**
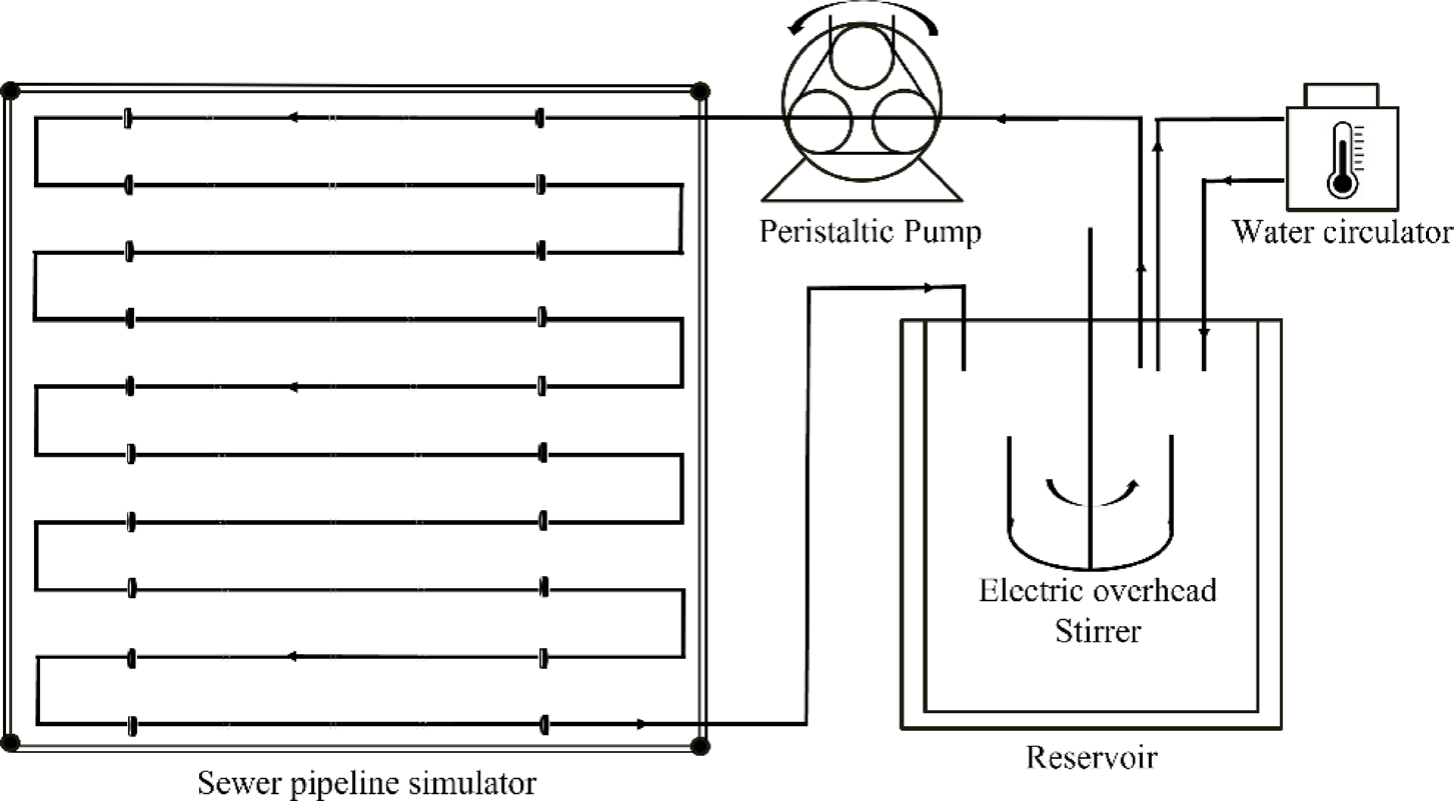
A schematic diagram of the lab-scale sewer pipeline simulator which is composed of wastewater reservoir, water circulator, peristaltic pump, and sewer pipeline simulator.

A laboratory-scale sewer pipeline simulator was designed to observe the impact of viral travel time in the sewer system on viral RNA degradation, as shown in Fig. 7. Wastewater influent from the WWTP was mixed in a reservoir (open bath) using an electric overhead stirrer (MS 5060D, MTOPS^®^, Korea) and maintained at 25 °C with a circulating bath. The operating temperature (25 °C) was selected as a mid-range setpoint to facilitate stable long-term operation and minimize temperature fluctuations during continuous circulation; it should not be interpreted as universally representative of sewer temperatures. The sewer pipeline simulator consisted of a ten-layered structure with 200 m of 8 mm diameter polyurethane tubes and a capacity of 15 L. Because the tubing did not provide intrinsic light shielding, the pipeline assembly was covered with an opaque light-shielding curtain during operation to minimize exposure to ambient light. A V6-6 L Peristaltic Pump (Baoding Shenchen Precision Pump Co., Ltd., Hebei, China) was used to circulate wastewater through a lab-scale sewer system.

### Operation of lab-scale sewer system

The lab-scale sewer system was operated separately with dechlorinated tap water and wastewater to evaluate viral RNA decay across water matrices. Before spiking, influent wastewater and dechlorinated tap water were tested as unspiked matrix controls by RT-qPCR, and endogenous HCoV-NL63 RNA was not detected. HCoV-NL63 (100 µL; initial concentration ~ 10¹⁰ GC mL⁻¹) was added to 50 L of tap water or wastewater and mixed for 30 min. The solution was circulated using a peristaltic pump at 50 mL min⁻¹ (3.0 L h⁻¹) for 6 h to prime the system and remove air bubbles (pipeline hold-up volume, 15 L), corresponding to ~ 18 L circulated during priming. After the pipeline was filled without bubbles, the system was operated continuously for 2 days (tap water) and 7 days (wastewater). The dechlorinated tap-water run was included as a low-matrix, low-biological-activity baseline to evaluate short-term RNA stability in the simulator under minimized biological and wastewater-matrix effects. This baseline run was intentionally limited to 2 days (48 h) as a preliminary stability check rather than a long-term decay experiment; therefore, the tap-water results are used to contextualize wastewater decay primarily within the shared observation window. These durations represent controlled observation windows for time-dependent decay under defined conditions and should not be interpreted as universal municipal sewer transit times^72^. The tap water run served as a low-biological-activity baseline and was therefore shorter; long-term tap water kinetics were not evaluated. For wastewater operation, the continuous experiment was divided into Stage I (0–7 days) and Stage II (7–13 days) based on the timing of spiking and partial replacement with fresh influent. On day 7, 35 L in the tanks was removed and replaced with an equal volume of freshly prepared influent wastewater. Because the pipeline hold-up volume was 15 L, complete replacement was not possible and ~ 15 L of Stage I wastewater remained in the pipeline after exchange. After refilling and thorough mixing, HCoV-NL63 was spiked to initiate Stage II (t = 0 for Stage II). Water samples (100 mL) were collected daily from the influent and outlet tanks and split into duplicates. For total cell counts, 1 mL aliquots were stored at 4 °C. For viral RNA analysis, samples were immediately frozen at − 80 °C until extraction; all stored samples were analyzed within one week of collection. Between experiments, tubing was disinfected by flushing with diluted sodium hypochlorite, followed by thorough rinsing with tap water to remove residual disinfectants before the next run.

### Analysis

#### Total cell number analysis

Flow cytometry was used to track and analyze changes in the total cell numbers in the lab-scale sewer system during operation, as previously described^102^. Briefly, wastewater samples from the lab-scale sewer system were collected daily and diluted 1:10 (v/v) with 1×PBS (100 µL sample mixed with 900 µL 1×PBS). Aliquot of SYBR Green I™ (10 µL) (×100 concentrate; Molecular Probes, Switzerland) which is a nucleic acid–specific fluorescent dye that binds only in the presence of nucleic acids and remains unbound when nucleic acids are absent was mixed with 1mL of each sample and incubated at 37 ℃ for 30 min. Total cell number was measured using CytoFLEX V3-B4-R3 Flow Cytometer (Beckman Coulter^®^, CA, USA), and results were analyzed using CytExpert software version 2.4.0.28 (Beckman Coulter^®^, CA, USA).

#### RNA extraction

For the batch test, total RNA was extracted from the samples using a modified Trizol-based extraction method^103^ which is cost-effective to deal with a number of samples made by batch-tests. Chloroform reagent (120 µL) was added to 200 µL samples containing 600 µL of Trizol^®^ reagent reacted for 15 min at room temperature (25 ℃). The chloroform-treated samples were centrifuged at 18,538 × g for 15 min. The supernatant was transferred to new 1.5 mL microtubes, and the same volume of 100% isopropanol as the supernatant was added and reacted for 10 min at RT. After centrifugation (18,538 × g, 10 min), the pellet was resuspended with 50 µL of diethylpyrocarbonate-treated water (DEPC-water) and incubated at 65 ℃ for 10 min. All RNA samples were stored at − 80 °C until further analysis. All samples were processed and RNA was extracted within one week of collection.

For the lab-scale sewer simulator test, Although the Maxwell^®^ RSC Enviro Nanotrap A TNA kit has a maximum processing volume of 10 mL, a modified version was used in this study to efficiently pre-treat 40 mL samples and extracted RNA with Maxwell^®^ RSC (Promega Corporation, Madison, WI) which is time-efficient and can be used with relatively small number of samples by lab-scale sewer simulator test. The concentration and purity of the extracted RNA were analyzed using a spectrophotometer (DeNovix DS-11, DE, USA). The extracted RNA was stored at − 80 ℃ until further analysis. All samples were processed and RNA was extracted within one week of collection.

#### RT-qPCR and digital droplet PCR (ddPCR) analysis

Quantification of NL63 gene concentrations was performed using RT-qPCR and/or RT-ddPCR with NL63 N-gene–specific primers (forward, 5′-GCGTGTTCCTACCAGAGAGGA-3′; reverse, 5′-GCTGTGGAAAAACCTTTGGCA-3′) and a probe (5′-ATGTTATTCAGTGCTTTGGTCCTCGTGAT-3′)^83^. RT-qPCR was the primary platform for routine quantification of experimental samples, whereas RT-ddPCR was used selectively for calibration/cross-validation via absolute quantification. For cross-validation, RT-ddPCR concentrations were aligned with RT-qPCR results using the same dilution-series framework and corresponding dilution factors.

For RT-qPCR, the PCR reaction mixture was prepared following the SensiFAST™ Probe No-ROX One-Step Kit’s guidelines (Bioline, Sydney, Australia). Briefly, the 20 µL mixture was composed of 10 µL of 2 ×SensiFAST Probe No-ROX One-Step Mix, 0.8 µL of 10 µM forward primer, 0.8 µL of 10 µM reverse primer, 0.2 µL of 10 µM probe, 0.2 µL of reverse transcriptase, 0.4 µL of RiboSafe RNase inhibitor, 3.6 µL of DEPC water, and 4.0 µL of RNA samples. The RT-qPCR protocol included an initial reverse transcription step at 45 °C for 10 min, followed by enzyme activation at 95 °C for 2 min and 40 amplification cycles (95 °C for 5 s and 60 °C for 20 s). The reaction was conducted using the AriaMx Real-Time PCR system (Agilent Technologies, CA, USA).

The RT-qPCR standard curve was generated using tenfold serial dilutions of HCoV-NL63 viral RNA (10⁰–10⁻⁸), analyzed in duplicate (*n* = 2). The regression equation was y = − 3.4087x + 9.8448 (R² = 0.9993). Negative controls (no-template controls and blank extraction controls) were included in all assays, and no amplification was observed. PCR inhibition was evaluated using a dilution-based test: a 10-fold dilution produced the expected ~ 3.3-cycle Ct shift, and RNA extracts showed comparable Ct shifts, indicating minimal inhibition. RT-qPCR LOD₉₅ and LOQ were determined from an eight-replicate tenfold dilution series, and both corresponded to dilution step 6 (8/8 positive; mean Ct = 32.35 ± 1.35, *n* = 8); higher dilutions showed inconsistent detection with late Ct values (Ct ≈ 36–38). Unspiked wastewater and tap-water matrix controls processed alongside study samples also showed no detectable amplification for HCoV-NL63.

For RT-ddPCR, assays were performed using the One-Step Advanced RT-ddPCR for Probes kit (Bio-Rad, Hercules, CA, USA) on a QX200 system. Reactions (22 µL) contained 5.5 µL supermix, 2.2 µL reverse transcriptase, 1.1 µL DTT (300 mM), 1 µL each of 20 µM forward and reverse primers, 0.3 µL of 20 µM probe, 9.9 µL PCR-grade water, and 5 µL RNA template. Droplets were generated using a QX200 droplet generator, and thermal cycling was: 25 °C for 3 min; 50 °C for 60 min; 40 cycles of 95 °C for 30 s and 55 °C for 1 min; hold at 4 °C. Because RT-ddPCR provides absolute quantification, a standard curve is not required. In this study, a tenfold dilution series (10⁻⁴–10⁻⁸) was analyzed by RT-ddPCR to characterize the working range (to avoid droplet saturation at high concentrations) and to support RT-qPCR cross-validation. For back-calculation, dilution step (x) was converted to copies L⁻¹ using y = 1 × 10¹³·exp(− 1.933x) and then to influent concentration using (20/4)×50/(0.2 × 1000); the ddPCR-equivalent LOD₉₅/LOQ corresponded to dilution step 6 (≈ 1.15 × 10⁸ copies L⁻¹ influent). Negative controls were included in all ddPCR runs, and no amplification was observed.

### Experimental standardization following MIQE guidelines

To ensure the reliability and comparability of RT-qPCR and RT-ddPCR results, experiments were conducted following standardized protocols in line with MIQE guidelines^104,105^. Due to the high number of samples analyzed, RNA purity was not assessed individually using Nanodrop or similar spectrophotometric methods; however, RNA extraction was performed using standardized methods specific to each experimental setup. For batch tests, RNA was extracted using Trizol-based extraction method, while for the lab-scale sewer simulator test, RNA extraction was carried out using Maxwell^®^ RSC (Promega Corporation, Madison, WI). By maintaining consistent protocols within each test type, variability was minimized. For selected samples, duplicate measurements were conducted to validate reproducibility. All RT-qPCR and ddPCR reactions were run using the same thermal cycler and standardized reaction conditions to reduce inter-run and inter-operator variability, respectively. Negative controls and non-template controls (NTCs) were included in every run to ensure specificity and to monitor contamination.

### Statistical analysis

The first-order decay rate constant (*k*) was calculated using Eq. (1) based on a first-order decay model^106^, In Eq. (1), C_t_ and C_0_ are genome-copy concentrations (GC) at time t and time 0, respectively, quantified primarily by RT-qPCR (with RT-ddPCR used for calibration/cross-validation as described above), and *k* is the decay rate constant^107^.

Because decay constants (*k*) were estimated from the relative change in measured RNA concentrations over time within an identical processing workflow, systematic losses during sample concentration and RNA extraction would scale both C_t_ and C_0_ similarly and thus do not materially affect *k*, provided recovery is approximately time-invariant within a condition. Accordingly, we report directly measured concentrations and do not apply a recovery-efficiency correction, consistent with recommendations that recovery controls be used for quality assurance and reported as metadata rather than used for numeric correction when proxy representativeness is uncertain^21,68^. Accordingly, no numerical recovery-efficiency correction was applied in estimating *k*.

1 $$\ln \left( {\frac{{C_{t} }}{{C_{0} }}} \right) = ~ - k \times t$$

The time for the viral RNA detected by RT-qPCR to decrease by 1 log (*T*_*90*_) is calculated as Eq. 2 below^31^.

2 $$T_{{90}} = \frac{{ - \ln \left( {0.1} \right)}}{k}$$

Statistical analysis was performed using GraphPad Prism 8.0.1 (GraphPad Software, La Jolla, CA, USA). Data are presented as mean ± standard deviation (SD). Differences between two independent groups were analyzed using an unpaired two-tailed Student’s t-test. One-way ANOVA followed by Tukey’s post hoc test was used to analyze differences among multiple groups. For all analyses, p-values < 0.05 were considered statistically significant.

## Supplementary Information

Below is the link to the electronic supplementary material.

Supplementary Material 1

## Data Availability

The data that support the findings of this study are available from the corresponding author upon reasonable request.
