# Supplementary material for: Environmental and microbial factors shaping SARS-CoV-2 RNA decay in wastewater: insights from batch tests and a lab-scale sewer pipeline simulator
Source: Sci Rep. 2026 Mar 19;16:14177. doi: 10.1038/s41598-026-44857-y (PMC13139382; doi:10.1038/s41598-026-44857-y)
Supplement: Supplementary file 1 — Supplementary Material 1 [file 41598_2026_44857_MOESM1_ESM.docx]

**Supplementary Information**

**Environmental and microbial factors shaping SARS-CoV-2 RNA decay in wastewater: insights from batch tests and a lab-scale sewer pipeline simulator**

JooAhn Jung^a,#^, Lan Hee Kim^b, #^, Sungpyo Kim^c,*^, Hyun Sik Jun^a,*^

^a^Department of Biotechnology and Bioinformatics, College of Science and Technology, Korea University, 2511 Sejong-ro, Sejong city, 30019, Republic of Korea

^b^Research Institute for Advanced Industrial Technology, Korea University, 2511 Sejong-ro, Sejong city, 30019, Republic of Korea

^c^Department of Environmental Systems Engineering, Korea University, 2511 Sejong-ro, Sejong city, 30019, Republic of Korea

^#^These authors contributed equally to this work and share the first authorship.

JooAhn Jung

Department of Biotechnology and Bioinformatics, College of Science and Technology, Korea University, 2511 Sejong-ro, Sejong city, 30019, Republic of Korea

E-mail: vtr2040@korea.ac.kr

Lan Hee Kim

Research Institute for Advanced Industrial Technology, Korea University, 2511 Sejong-ro, Sejong city, 30019, Republic of Korea

E-mail: lanheekim@korea.ac.kr

^*^Corresponding author.

Sungpyo Kim

Department of Environmental Systems Engineering, Korea University, 2511 Sejong-ro, Sejong city, 30019, Republic of Korea

E-mail: [ub1905ub@korea.ac.kr](mailto:ub1905ub@korea.ac.kr)

Hyun Sik Jun

Department of Biotechnology and Bioinformatics, College of Science and Technology, Korea University, Sejong, 30019, Republic of Korea

E-mail: [toddjun@korea.ac.kr](mailto:toddjun@korea.ac.kr)


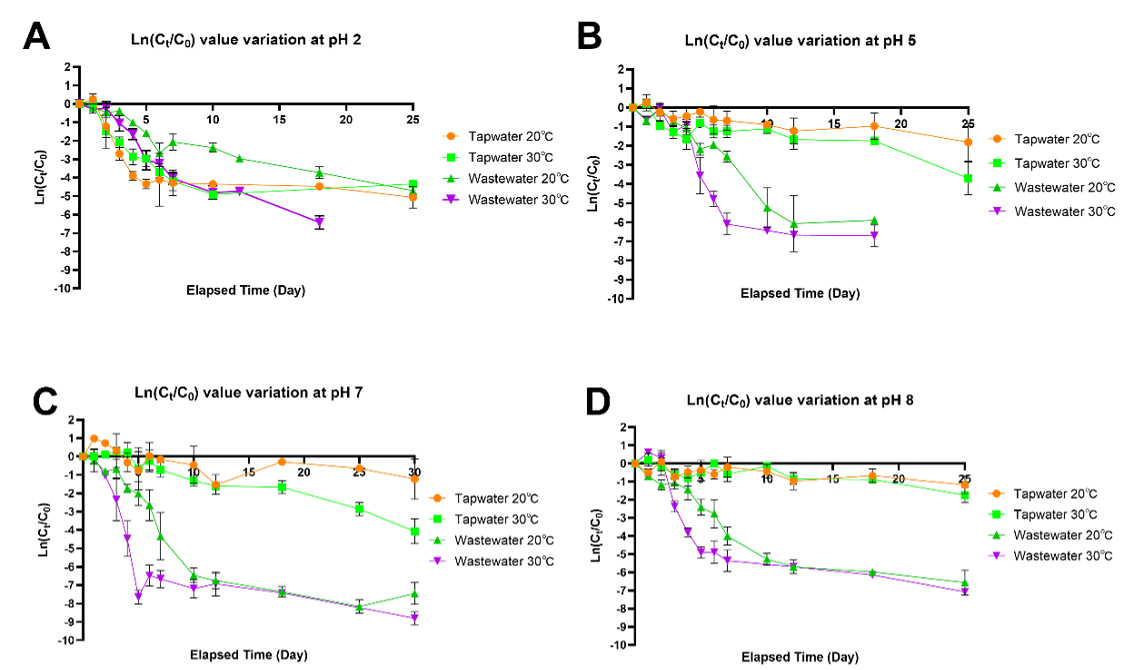


Supplementary Figure 1. Assessment of HCoV-NL63 virus decay rates under varying physicochemical conditions. (A)–(D) depict the decay rates of HCoV-NL63 in dechlorinated tap water and wastewater, adjusted to pH levels of 2, 5, 7, and 8, at temperatures of 20 °C and 30 °C, over a 25-day period to comprehensively analyze viral stability in different environmental matrices.

**Supplementary Table 1.** Viral decay rate in wastewater influent according to Microbial Concentration as biological Factors for 15 days of incubation

| **Water Matrix** | **Temperature (℃)** | **NL63 virus decay rate** | |
| --- | --- | --- | --- |
|  |  | **ln(C_t_/C_0_)** | ***k*(d^-1^)** |
| Raw wastewater (WW) | 20 | -4.10 (± 0.21) | 1.72 |
|  | 30 | -6.90 (± 0.34) | 1.73 |
| 10^-2^ diluted WW | 20 | -1.83 (± 0.26) | 0.92 |
|  | 30 | -1.78 (± 0.21) | 1.34 |
| 10^-3^ diluted WW | 20 | -0.98 (± 0.51) | 0.33 |
|  | 30 | -0.79 (± 0.15) | 0.27 |
| 10^-4^ diluted WW | 20 | -0.76 (± 0.18) | 0.25 |
|  | 30 | -1.52 (± 0.22) | 0.51 |

**Supplementary Table 2.** Viral decay rate in wastewater influent according to different concentration of Suspended Solids for 24 h of incubation

| **SS mg/L** | **Temperature (℃)** | **NL63 virus decay rate** | |
| --- | --- | --- | --- |
|  |  | **ln(C_t_/C_0_)** | ***k*(d^-1^)** |
| 216 | 20 | -1.28 (± 0.19) | 1.28 |
|  | 30 | -4.00 (± 0.27) | 4.00 |
| 134 | 20 | -0.59 (± 0.11) | 0.59 |
|  | 30 | -3.11 (± 0.41) | 3.11 |
| 74 | 20 | 0.30 (± 0.13) | -0.30 |
|  | 30 | -2.77 (± 0.60) | 2.77 |
| Blank | 20 | 1.00 (± 0.56) | -1.00 |
|  | 30 | -1.52 (± 0.22) | -0.37 |

**Supplementary Table 3.** Viral decay rate in wastewater influent by Suspended Solids and Microbes in wastewater for 30 days of incubation at 30 ℃

| **Water Matrix** | **NL63 virus decay rate** | |
| --- | --- | --- |
|  | **ln(C_t_/C_0_)** | ***k*(d^-1^)** |
| Raw wastewater | -7.06 (± 0.95) | 1.77 |
| GF/C filtered wastewater | -4.48 (± 0.26) | 1.12 |
| GF/C filtered and 0.1% NaN_3_-treated wastewater | -1.04 (± 0.22) | 0.52 |
| 0.1% NaN_3_-treated wastewater | -2.81 (± 0.58) | 0.47 |
|  |  |  |

**Supplementary Table S4.** Viral decay rate in wastewater influent by Lab-scale sewer pipeline system for 23 days of incubation at 25 ℃

| **Water Matrix** | **NL63 virus decay rate** | |
| --- | --- | --- |
|  | **ln(C_t_/C_0_)** | ***k*(d^-1^)** |
| Raw wastewater | -7.06 (± 0.95) | 1.77 |
| GF/C filtered wastewater | -4.48 (± 0.26) | 1.12 |
| GF/C filtered and 0.1% NaN_3_-treated wastewater | -1.04 (± 0.22) | 0.52 |
| 0.1% NaN_3_-treated wastewater | -2.81 (± 0.58) | 0.47 |

**Supplementary Table 5.** Comparison of qPCR Ct values for HCoV-NL63 stock Before and After long-term storage at -80 ℃

| **Storage Condition** | **Ct (Mean ± SEM)** | **t-value** | **p-value** | **95% CI** |
| --- | --- | --- | --- | --- |
| Before storage | 10.42 ± 0.20 | - | - | - |
| After 1 year at -80 ℃ | 10.77 ± 0.15 | 1.456 | 0.2827 | -0.6844 to 1.384 |

Abbreviations: HCoV-NL63 = Human Coronavirus NL63, SEM = Standard Error of the Mean, CI = Confidence Interval.
